# Prevalence and related factors of common mental disorders during pregnancy in Japan: a cross-sectional study

**DOI:** 10.1186/s13030-016-0069-1

**Published:** 2016-05-20

**Authors:** Kentaro Usuda, Daisuke Nishi, Miyuki Makino, Hisateru Tachimori, Yutaka Matsuoka, Yo Sano, Takako Konishi, Tadashi Takeshima

**Affiliations:** Toda Chuo Women’s Hospital, 2-26-3 Kamitoda, Toda, Saitama 335-0022 Japan; Tokyo Medical University, 6-7-1 Nishishinjuku, Shinjuku-ku, Tokyo, 160-0023 Japan; Department of Mental Health Policy and Evaluation, National Institute of Mental Health, National Center of Neurology and Psychiatry, 4-1-1 Ogawa-Higashi, Kodaira, Tokyo, 187-8553 Japan; Center for Public Health Sciences, National Cancer Center, 5-1-1 Tsukizi, Chuo-ku, Tokyo, 104-0045 Japan; National Center for Cognitive Behavior Therapy and Research, National Center of Neurology and Psychiatry, 4-1-1 Ogawa-Higashi, Kodaira, Tokyo, 187-8553 Japan; Musashino University, 3-3-3 Ariake, Koto-ku, Tokyo, 135-8181 Japan; Health and Social Welfare Bureau, Kawasaki City Office, 3-16-1 Ida, Nakahara-ku, Kawasaki, Kanagawa 211-0035 Japan

**Keywords:** Pregnancy, Prevalence, Common mental disorders, Structured interview

## Abstract

**Background:**

Common mental disorders (CMD) during pregnancy can have a clearly harmful influence on both mothers and children. Some studies have reported related factors for mental disorders, such as region-specific background. This study examined the prevalence of CMD and its related factors in mid-pregnancy in Japan.

**Methods:**

Pregnant women between 12 and 24 weeks gestation and aged ≥20 years were consecutively recruited at a maternity hospital in Japan between May 2014 and September 2014. CMD were diagnosed using the Mini-International Neuropsychiatric Interview (MINI), self-rated depressive symptoms were assessed using the Edinburgh Postnatal Depression Scale, and interpersonal traumatic experience was measured using the Life Events Checklist.

**Results:**

Among 297 eligible pregnant women, 177 participated in the study. Two participants (1.1 %) met the criteria for major depressive disorder. The most frequent diagnosis was agoraphobia (*n* = 7; 3.9 %). Eleven participants (6.2 %) met the criteria for one or more diagnoses, with 2 participants having two mental disorders and 3 having three mental disorders. Six participants developed CMD after gestation. Logistic regression analysis revealed history of psychiatric disorder, past interpersonal traumatic experience, and feeling pressure to have a child were associated with CMD.

**Conclusion:**

These findings indicate a lower prevalence of CMD in mid-pregnancy in Japan than reported in most other countries. Besides the related factors reported previously, feeling pressure to have a child might increase risk for CMD among pregnant women in Japan. Asian cultural background might be related to the lower CMD prevalence and risk factors identified in this study.

## Background

Depression during pregnancy has widespread negative effects on mothers and babies including increased risks for preterm birth, low birth weight, postpartum depression, self-harm or suicide, failure to seek prenatal care, difficulty performing usual activities, poor diet, and tobacco and alcohol use [1, 2]. The effects of common mental disorders (CMD), which usually include not only mood disorders but anxiety and substance abuse disorders, on both pregnant women and their babies have also drawn attention. For instance, posttraumatic stress disorder (PTSD) during pregnancy was recently shown to increase the risk for preterm birth [3]. Prevalence of antenatal depression varies between 5.2 and 17.8 % worldwide [4–9] and for anxiety disorder varies between 0.0 and 10.5 % [3, 5, 7–11].

Sociocultural as well as methodological differences might account for some of the variance. It has been suggested that Asian populations report symptoms less often partly due to stigma [12, 13] and that some of the risk factors for depression and CMD might be relatively uncommon in Asia [14]. As an example, postpartum depression was more prevalent among women in India and China who gave birth to a girl than those who gave birth to a boy [15, 16], but no such trend was found among women in Western countries [17]. According to some researchers, the source of the still deeply ingrained gender preference in favor of boys in Asia is that male children carry on the family name, the family business, and contribute financially to the family [16, 18].

For over 150 years, Japan has been greatly affected by Western culture, while at the same time has retained its cultural identity in many ways. It would be highly relevant, therefore, to examine from a sociocultural perspective the prevalence and risk factors for CMD during pregnancy in Japan. While the prevalence and risk factors for CMD among pregnant women expecting their first babies have been investigated in a study involving clinics affiliated with medical universities [5], the findings might not generalize to pregnant women with children. Moreover, fewer than 10 % of pregnant women give birth at university hospitals in Japan [19, 20], and women who do so might have different characteristics from the majority of women who give birth in non-university hospital clinics.

This study aimed first to clarify prevalence and related factors of CMD by conducting a standardized structured interview among pregnant women in a local hospital, and second to compare these findings with those in other countries.

## Methods

### Participants

Participants were consecutively recruited from Tuesday to Friday between May 2014 and September 2014 at Toda Chuo Women’s Hospital (TCWH), Saitama Prefecture, which is located in the greater Tokyo area. Recruitment was not carried out on Mondays due to schedule conflicts among the research team, however, pregnant women who visited TCWH on Mondays did not differ significantly in terms of age or Edinburgh Postnatal Depression Scale (EPDS) scores from those who attended on other days of the week. Eligibility criteria for all pregnant women were: (1) 12–24 weeks of gestation, (2) age ≥20 years, and (3) ability to read and write Japanese. Based on previous studies [21, 22], we estimated that 6 % of pregnant women would have EPDS scores of ≥9, with a positive predictive value of 50 %; thus, prevalence of depression would be 3 %. We also estimated that width of the 95 % confidence interval (CI) would be 3 % with a power of 0.6. Therefore, 173 participants would be needed for the study.

**Fig. 1:**
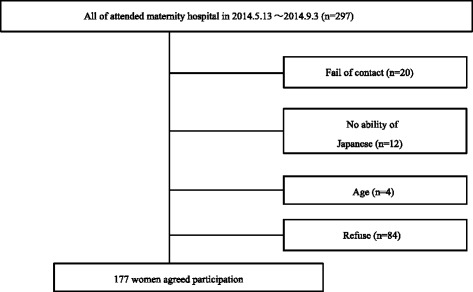
Flow chart of the study

Figure 1 shows the flowchart of recruitment. From May 2014 to September 2014, of 277 women approached out of 297 potentially eligible women, 20 were failed to contact, 12 had no ability of Japanese, and 4 (aged < 20 years) were excluded. Among 261 women, 177 (67.8 %) agreed to participate in the study.

### Measures and procedures

This study was conducted with the approval of the Institutional Review Board of TCWH. All pregnant women who attended TCWH during the study period were asked to take the EPDS and received a brief explanation of the study. Eligible women were provided a complete description of the study during their next visit, at which time written informed consent was obtained. After the assessment, each participant received a gift voucher (2,000 JPY [17 USD]) for their participation.

A questionnaire inquired about participants’, gestational age, age, education level, work status, household income in one year, marriage status, smoking status (never, current, or past), number of family members, planned pregnancy (yes, no), pressures related to having a child (yes, no), past psychiatric history (yes, no), family psychiatric history (yes, no), and childbirth experience (primipara or unipara/multipara).

The Mini-International Neuropsychiatric Interview (MINI) [23] is a brief (15–30 min) structured interview widely used to evaluate the presence of Axis I psychiatric disorders, as defined by the Diagnostic and Statistical Manual of Mental Disorders, Fourth Edition [24]. Assessment in this study included the following psychiatric disorders: major depressive episodes, dysthymia, manic and hypomanic episodes, generalized anxiety disorder, panic disorders, agoraphobia, obsessive-compulsive disorder, posttraumatic stress disorder, bulimia and anorexia nervosa, and alcohol or other substance dependence, which were defined as CMD in the study. The MINI was conducted by three trained examiners, namely, a trained psychiatrist (DN), who trained two clinical psychologists (KU, MM) through lectures and role playing. To assess inter-rater reliability, the three raters (one conducting the interview and one observing) assessed a random sample of 10 cases. Ratings were reliable for all diagnoses without psychotic disorders, with kappa values of 1.0.

Self-report depressive symptoms were obtained using the EPDS, which is used most often for screening perinatal depression in international research. This self-report measure focuses on cognitive symptoms of depression, excluding somatic items that can generate false positives during pregnancy and postpartum [25]. The EPDS, which has been validated in Japan [22], comprises 10-items, scored 0–3 points per item for a potential scale score of 0–30.

The Life Events Checklist, which was developed alongside with the Clinician-Administered PTSD Scale, was used to assess interpersonal traumatic experience [26, 27]. Participants were asked if they had ever experienced an interpersonal traumatic event known to potentially cause PTSD in their lifetime, such as interpersonal violence, weapon assault, confinement, sexual violence, other harmful sexual experience, and physical abuse.

### Statistical analysis

We calculated the prevalence (95 % CI) of CMD. To establish a model of factors related to CMD, we selected the following factors as basic background information: age, work status, and family psychiatric history. Work status was coded as 0 (part-time, not working, or student) or 1 (working full-time), and family psychiatric history as 0 (no history of psychiatric hospitalization (awareness of problem but no visit) or 1 (apparent mental symptoms).

Psychiatric history [7, 28], primipara [11], past interpersonal traumatic experiences [11, 28], work status and feeling pressure to have a child were selected because previous studies showed these variables were associated with CMD during pregnancy. Regarding work status, having regular jobs might be protective for depressive symptoms [29, 30].

Also, there seems to be the common wish in Asia to have a male child to carry on the family name. Feeling pressure to have a child is particularly relevant for pregnant women between 12 and 24 weeks of gestation who might not yet know the sex of the fetus. Additionally, marriage status [7], home crowding [9], number of children [7] and alcohol consumption [9] are reported to be factors related to mental disorder or current suicide risk. However, we excluded these factors from analysis because each included <10 participants in this study.

We performed bivariate and multivariable logistic regression analyses to identify independent significant factors associated with CMD during pregnancy, and reported odds ratios and 95 % CIs. All statistical analysis was performed using SPSS statistical software, version 22.0 for Windows (IBM Corporation, Armonk, NY). All tests were two-sided, and *p*-values ≤0.05 were considered statistically significant.

## Results

### Prevalence of CMD

The demographic characteristics are shown in Table 1. Table 2 shows CMD prevalence in the second trimester. Two participants (1.1 %) met the criteria for major depressive disorder. The most frequent diagnosis was agoraphobia (*n* = 7; 3.9 %). Also, 11 (6.2 %) participants met the criteria for one or more diagnoses: 2 had two mental disorders and 3 had three mental disorders. Six women of the 11 women developed CMD after gestation.

**Table 1:** Demographic and characteristics of pregnant women who participated in the study

Variables	Number	Percent	Mean	SD
Gestational age (week)	177		17.26	1.707
Age	177		31.15	4.378
Education				
Junior high school	5	2.8		
High school	37	20.9		
Junior or technical college	67	37.9		
University or more	68	38.4		
Work				
No job or part time	116	65.5		
Full time job	61	34.5		
Household income				
< 3million yen	14	7.9		
3million yen ~ 5million yen	55	31.1		
5million yen ~ 7million yen	61	34.5		
≧7million yen	47	26.6		
Marriage status				
Marriage	169	95.5		
Commuter marriage	1	0.6		
Cohabitation	1	0.6		
Remarriage	1	0.6		
Going to get Married	5	2.8		
Number of family members				
1 person	1	0.6		
2 persons	96	54.2		
3 persons ≦	80	45.2		
Smoke				
Never smoke	133	75.1		
Current smoke	0	0		
past smoke	44	24.9		
Birth experience				
Primipara	97	54.8		
Unipara or Multipara	80	45.2		
Planned pregnancy, yes	117	66.1		
Pressure to give birth to child, yes	28	15.8		
Past psychiatric history, yes	20	11.3		
Past psychiatric history of family, yes	15	8.5		
Interpersonal traumatic experience, yes	16	9.0		
EPDS score	177		2.82	3.052

**Table 2:** Prevalence of psychiatric diagnosis at gestational age 12 weeks to 24 weeks in a local maternity hospital (Total = 177)

Diagnosis	Number	% (95 % CI)
Mood disorders		
Major depression	2	1.1 (0.00–0.04)
Dysthymia	0	0.0 (0.00–0.00)
Manic episode	0	0.0 (0.00–0.00)
Anxiety disorders		
Panic disorder	2	1.1 (0.00–0.04)
Agoraphobia	7	3.9 (0.02–0.08)
Social anxiety disorder	2	1.1 (0.00–0.04)
Generalized anxiety disorder	3	1.7 (0.01–0.05)
Obsessive-compulsive disorder	1	0.6 (0.00–0.03)
PTSD (Post traumatic stress disorder)	0	0.0 (0.00–0.00)
Substance use disorders		
Alcohol dependence	2	1.1 (0.00–0.04)
Alcohol abuse	0	0.0 (0.00–0.00)
Drug dependence	0	0.0 (0.00–0.00)
Drug abuse	0	0.0 (0.00–0.00)
Eating disorders		
Anorexia nervosa	0	0.0 (0.00–0.00)
Bulimia nervosa	0	0.0 (0.00–0.00)
At least one diagnosis	11	6.2 (0.03–0.11)

### Related factors for CMD

Table 3 gives the results of bivariate and multivariate logistic regression analysis, with CMD as a dependent variable. In bivariate logistic regression analysis, CMD was significantly associated with younger age, past psychiatric history, family psychiatric history, planned pregnancy, and interpersonal traumatic experience In multivariate logistic regression analysis, CMD was significantly associated with psychiatric history, interpersonal traumatic experience, and feeling pressure to have a child.

**Table 3:** The result of logistic bivariate and multivariate regression analyses (*n* = 177)

Variables	Bivariate	*P*	Multivariate	*P*
	OR (95 % CI)		OR (95 % CI)	
Age	0.32 (0.04–2.54)	0.28	0.80 (0.65–1.00)	0.04
Full time work, yes	0.40 (0.08–1.93)	0.26	0.12 (0.01–1.54)	0.10
Past psychiatric history, yes	13.03 (3.53–48.14)	<0.01**	6.80 (1.41–32.52)	0.02*
Family past psychiatric history, yes	4.81 (1.13–20.54)	0.03*	6.27 (0.94–41.80)	0.06
Birth experience, yes	0.25 (0.05–1.20)	0.08	0.44 (0.06–3.56)	0.44
Past interpersonal traumatic experiences, yes	7.33 (1.88–28.62)	<0.01**	10.91 (1.61–74.10)	0.01*
Pressure to give birth to child, yes	3.38 (0.92–12.44)	0.07	11.56 (1.47–91.02)	0.02*

*OR* odds ratio, *CI* confidence interval

**p < 0.01、*p < 0.05

## Discussion

Prevalence of major depressive disorder (MDD) and CMD was 1.1 and 6.2 %, respectively, which are much lower figures than seen in other countries [3, 6–11]. Such prevalence was comparable to the point prevalence in the general population in Japan [31]. It is well known that prevalence of CMD is in general lower in Asia than in other regions [32]. Aside from the stigma attached to having CMD, people in Asia seek treatment only when their symptoms cause significant impairment [12], which could reduce symptom reporting. Also, some researchers have suggested that the prevalence rate of PTSD might be related to the infant mortality rate, which is a sign of social circumstance and basic population health [33]. These cultural factors might explain the low prevalence of CMD we found among pregnant women in Japan. This low prevalence is also lower than that found for MDD (5.6 %) and CMD (12.1 %) among women in Japan expecting their first babies in clinics affiliated with medical universities [5]: some of the pregnant women who had visited university hospitals might have received fertility treatment or had complications, which also could account for the higher reported prevalence.

Feeling pressure to have a child was significantly associated with CMD, and might reflect traditional beliefs still present in Japan, where continuity of the family name is greatly valued. A previous study in Vietnam showed that strong gender preference increased the risk for CMD, although such women with CMD were likely to recover before late pregnancy [34]. On the other hand, dissatisfaction with a newborn baby’s sex was shown to be associated with postnatal depression among Japanese pregnant women who had their first baby [5]. Whether the impact of feeling pressure to have a child predicts depression in late pregnancy or after childbirth should be further elucidated.

There could be another cultural explanation why feeling pressure to have a child was associated with CMD. Many Asian cultures, including Japanese culture, emphasize connecting to, attending to, and fitting in with others [35]. Other people are critical for self-validation in these interdependent cultures, and people are motivated to find a way to fit in with relevant others, which sometimes engenders a sense of obligation [35]. The goals of relevant others are sometimes experienced as one’s personal goals, or meeting others’ goals might be a necessary requirement for satisfying one’s own goals [35]. Thus, some pregnant women might feel stressed when they compare themselves to other women who already have children or when faced with the high expectations of their husbands and in-laws that they produce a child without any complications.

Although unplanned pregnancy was not associated with CMD in this study, many previous studies have shown an association [7, 11, 28, 36], which might be due to Japan’s relatively high rate of elective abortions [37]. In the present study, we recruited only women with planned pregnancies; we did not recruit those with unplanned pregnancies who chose to terminate their pregnancy, which might have affected the results.

Past interpersonal traumatic experience was found to be significantly associated with CMD, which is consistent with the findings of previous studies [9, 28, 34]. Past interpersonal traumatic experience is past traumatic events in their lifetime, such as interpersonal violence, weapon assault, confinement, sexual violence, other harmful sexual experience, and physical abuse. Not surprisingly, past psychiatric history was associated with CMD. It is well known that the prevalence of CMD and suicidal behavior is higher in women who have been exposed to gender-based violence [38]. Therefore, clinicians should be alert to pregnant women’s past traumatic experience as well as past psychiatric history.

Younger age almost reached statistical significance. Findings of previous studies on age have been inconsistent, with older age shown to be associated with CMD in Vietnam [11, 34] but found to be protective for CMD in Japan [5] and the United States [39]. In Vietnam, women usually marry in their early 20's and have their first baby by age 25. Women over age 30 who are having a first or second baby might reflect difficulties establishing a partnership due to stigmatizing circumstances such as personal or family mental health problems, extreme poverty, or fertility problems [34]. However, in countries without these cultural circumstances, older age might not be a risk factor for CMD among pregnant women. In Japan, the trend is towards having children later in life [40], with no stigma against relatively older pregnant women. Also, being older is sometimes associated with economic stability or increased resilience to stressful events.

The strengths of this study are the use of reliable and standardized assessments and consecutive sampling. However, there are several limitations. First, approximately 30 % of pregnant women who refused to participate in the study had EPDS scores that were significantly higher than those of the participants, suggesting that they might be burdened further by participating. Therefore, prevalence might be underestimated in this study. Second, a relatively small number of women met the diagnostic criteria of various psychiatric disorders, although data from participants consecutively recruited in this study might provide valuable additional evidence to that of previous studies where participants were not recruited consecutively. Third, study duration was relatively short (i.e., May 2014 to September 2014), which introduces an additional factor of winter seasonal affective disorder, a recurrent subtype of depression [41]. This factor might also lead to underestimated prevalence. Fourth, we did not analyze important factors such as marital status [7] or home crowding [9]. Finally, our results were obtained from one hospital in the greater Tokyo area and thus the findings of this study might not generalize to other regions in Japan.

## Conclusion

Our findings suggest that prevalence of CMD during mid-pregnancy in Japan is lower than that in most other countries. In addition to risk factors shown previously, feeling pressure to have a child might increase the risk for CMD, which might reflect certain aspects of Japanese culture.

### Consent for publication

This study was conducted with the approval of the Institutional Review Board of Toda Chuo Women’s Hospital. All pregnant women who attended TCWH during the study period were asked to take the EPDS and received a brief explanation of the study. Eligible women were provided a complete description of the study during their next visit, at which time written informed consent was obtained. After the assessment, each participant received a gift voucher (2,000 JPY [17 USD]) for their participation.
