# Erratum to: Prevalence and related factors of common mental disorders during pregnancy in Japan: a cross-sectional study

**DOI:** 10.1186/s13030-016-0077-1

**Published:** 2016-09-05

**Authors:** Kentaro Usuda, Daisuke Nishi, Miyuki Makino, Hisateru Tachimori, Yutaka Matsuoka, Yo Sano, Takako Konishi, Tadashi Takeshima

**Affiliations:** 1 Toda Chuo Women’s Hospital, 2-26-3 Kamitoda, Toda, Saitama 335-0022 Japan; 2 Tokyo Medical University, 6-7-1 Nishishinjuku, Shinjuku-ku, Tokyo 160-0023 Japan; 3 Department of Mental Health Policy and Evaluation, National Institute of Mental Health, National Center of Neurology and Psychiatry, 4-1-1 Ogawa-Higashi, Kodaira, Tokyo 187-8553 Japan; 4 Center for Public Health Sciences, National Cancer Center, 5-1-1 Tsukizi, Chuo-ku, Tokyo 104-0045 Japan; 5 National Center for Cognitive Behavior Therapy and Research, National Center of Neurology and Psychiatry, 4-1-1 Ogawa-Higashi, Kodaira, Tokyo 187-8553 Japan; 6 Musashino University, 3-3-3 Ariake, Koto-ku, Tokyo 135-8181 Japan; 7 Health and Social Welfare Bureau, Kawasaki City Office, 3-16-1 Ida, Nakahara-ku, Kawasaki, Kanagawa 211-0035 Japan

## Erratum

After publication of the original article [1], the authors noticed there was an error to the values for “Generalized anxiety disorder”, “Obsessive-compulsive disorder” and “PTSD (Post traumatic stress disorder)” listed underneath the “% (95% CI)” column. The correct version of Table 2, is included in this erratum.

**Table 2:** Prevalence of psychiatric diagnosis at gestational age 12 weeks to 24 weeks in a local maternity hospital (Total = 177)

Diagnosis	Number	% (95 % CI)
Mood disorders		
Major depression	2	1.1 (0.00–0.04)
Dysthymia	0	0.0 (0.00–0.00)
Manic episode	0	0.0 (0.00–0.00)
Anxiety disorders		
Panic disorder	2	1.1 (0.00–0.04)
Agoraphobia	7	3.9 (0.02–0.08)
Social anxiety disorder	2	1.1 (0.00–0.04)
Generalized anxiety disorder	0	0.0 (0.00–0.00)
Obsessive-compulsive disorder	3	1.7 (0.01–0.05)
PTSD (Post traumatic stress disorder)	1	0.6 (0.00–0.03)
Substance use disorders		
Alcohol dependence	2	1.1 (0.00–0.04)
Alcohol abuse	0	0.0 (0.00–0.00)
Drug dependence	0	0.0 (0.00–0.00)
Drug abuse	0	0.0 (0.00–0.00)
Eating disorders		
Anorexia nervosa	0	0.0 (0.00–0.00)
Bulimia nervosa	0	0.0 (0.00–0.00)
At least one diagnosis	11	6.2 (0.03–0.11)
